# Accumulation of Endogenous LITAF in Aggresomes

**DOI:** 10.1371/journal.pone.0030003

**Published:** 2012-01-19

**Authors:** Heather E. Eaton, Julie Metcalf, Andressa Ferreira Lacerda, Craig R. Brunetti

**Affiliations:** Department of Biology, Trent University, Peterborough, Ontario, Canada; University of South Florida College of Medicine, United States of America

## Abstract

LITAF is a 161 amino acid cellular protein which includes a proline rich N-terminus and a conserved C-terminal domain known as the simple-like domain. Mutations in LITAF have been identified in Charcot-Marie tooth disease, a disease characterized by protein aggregates. Cells transfected with cellular LITAF reveal that LITAF is localized to late endosomes/lysosomes. Here we investigated the intracellular localization of endogenous LITAF. We demonstrated that endogenous LITAF accumulates at a discrete cytoplasmic site in BGMK cells that we identify as the aggresome. To determine the domain within LITAF that is responsible for the localization of LITAF to aggresomes, we created a construct that contained the C-terminal simple-like domain of LITAF and found that this construct also localizes to aggresomes. These data suggest the simple-like domain is responsible for targeting endogenous LITAF to the aggresome.

## Introduction

Lipopolysaccharide-induced tumor necrosis factor-alpha factor (LITAF) is a small cellular protein comprised of 161 amino acids with a currently unknown function [1]. LITAF is composed of two very distinct termini. The N-terminus is proline rich and contains proline rich binding sites (PPXY, (P(S/T)AP) for several proteins including the E3 ligases neuronal precursor cell expressed developmentally downregulated 4 (Nedd4) [2], [3], [4], Itch [2], [3], [4], the E2 ubiquitin conjugating enzyme tumor suppressor gene 101 (TSG101) [3], and the putative tumor suppressor WW domain oxidoreductase (WWOX) [5]. The C-terminus of LITAF is cysteine rich and contains a C3H4-type zinc finger domain interrupted by a stretch of 23 hydrophobic amino acids [1]. This unique domain is termed the simple-like domain (SLD) and is highly conserved throughout many eukaryotes. The SLD also contains a YXXø (where ø is any hydrophobic amino acid) and a dileucine motif [1]. Proteins containing YXXø motifs interact with clathrin adaptor complexes to sort and target membrane proteins throughout endosomes, the Golgi network, and lysosomes [6], [7]. Furthermore, proteins containing dileucine motifs are also commonly targeted to the endosome/lysosome network.

Although the cellular localization of LITAF appears to be inconsistent between different cell types, its localization appears consistently along the pathway of lysosomal degradation. Ectopically expressed LITAF localizes within late endosomes/lysosomes in BGMK, HEK 293T, COS-7, and THP-1 cell lines [1], [4], the Golgi apparatus in HEK 293T and MCF-7 cells [3], [5], as well to the plasma membrane in HEK 293T cells [3]. Endogenous LITAF has only been reported in B lymphoblastoid cells where its intracellular localization was not determined [3].

Our previous research revealed that recombinant LITAF localized to the late endosome/lysosomes in BGMK cells [4]. Since the localization of endogenous LITAF has not been reported, we decided to investigate the cellular localization of endogenous LITAF in BGMK cells.

## Results

### Endogenous LITAF localizes to a perinuclear region within the cell

**Figure 1:**
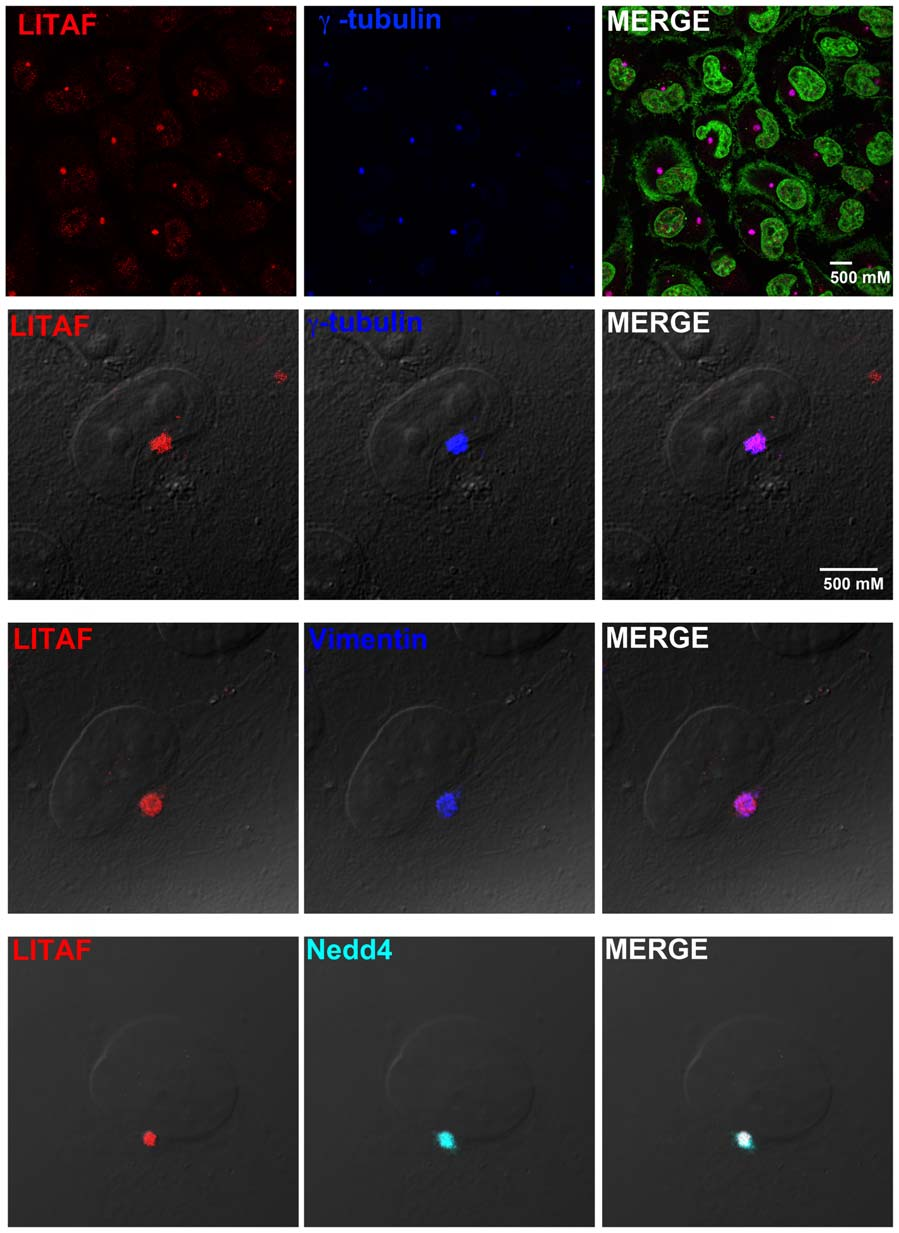
Endogenous LITAF accumulates in aggresomes. In order to detect endogenous LITAF, BGMK cells were fixed and indirect immunofluorescence was performed. Anti-LITAF antibodies were used to detect endogenous LITAF (red), anti-Nedd4 antibodies were used to detect Nedd4 (cyan), and anti-vimentin or anti-γ-tubulin antibodies were visualized (blue) to determine localization of LITAF. Nuclei were visualized using differential interference contrast (DIC) or ToPro (green). All images were taken using a laser scanning confocal microscope.

In order to determine cellular localization of endogenous LITAF, BGMK cells were fixed and LITAF was detected using a mouse polyclonal anti-LITAF antibody. We were able to detect endogenous LITAF in BGMK cells (Figure 1). However, we were unable to detect endogenous LITAF in a variety of other cell cells lines such as HEK-293T, Hela cells, or primary neurons (data not shown).

Endogenous LITAF exhibited concentrated perinuclear staining, which co-localized with γ-tubulin (Figure 1) in all cells examined. γ-tubulin is a highly conserved protein found in the microtubule organizing center (MTOC). In higher eukaryotes, the MTOC, or centrosome, is composed of a pair of centrioles embedded in a matrix of pericentriolar material (which includes γ-tubulin) [8]. The region of the cell that contains the MTOC is also the site of the aggresome. Aggresomes are pericentriolar subcellular structures encapsulated in a vimentin sheath that contain aggregated misfolded ubiquitinated proteins [9], [10], [11]. Aggresomes are formed when the degradation capacity of the ubiquitin-proteasome system is overwhelmed and misfolded proteins are transported from the periphery of the cell to proteasomes that are located adjacent to the MTOC [9], [10], [11].

Since recombinant LITAF is localized to the late endosome/lysosome, the site of protein degradation in the cell, we tested whether LITAF co-localized with aggresomes, another site of protein degradation in the cell. Since the aggresomes are adjacent to the MTOC, it is possible that LITAF is localized to the aggresome rather than the MTOC. To test this hypothesis, we examined the localization of LITAF with respect to the aggresome marker, vimentin [9], [10], [11]. In BGMK cells, LITAF co-localizes with vimentin in all cells examined (Figure 1). Localization of LITAF to the pericentriolar region and with vimentin suggests that LITAF localizes to a perinuclear region consistent with the aggresome.

### Endogenous Nedd4 and LITAF co-localize at a perinuclear site

Endogenous LITAF localizes to the MTOC/aggresome in BGMK cells. Nedd4, an E3 ubiquitin ligase is a known binding partner of LITAF [2], [3]. We wanted to determine whether endogenous Nedd4 co-localized with LITAF. In order to determine whether Nedd4 co-localizes along with LITAF, immunofluorescence was performed using both anti-LITAF and anti-Nedd4 antibodies. We found that endogenous LITAF and Nedd4 co-localized in every cell that expressed LITAF (Figure 1). These data therefore suggest that endogenous LITAF is localized to the MTOC/aggresome along with Nedd4 in BGMK cells.

### Endogenous LITAF localizes to the aggresome

**Figure 2:**
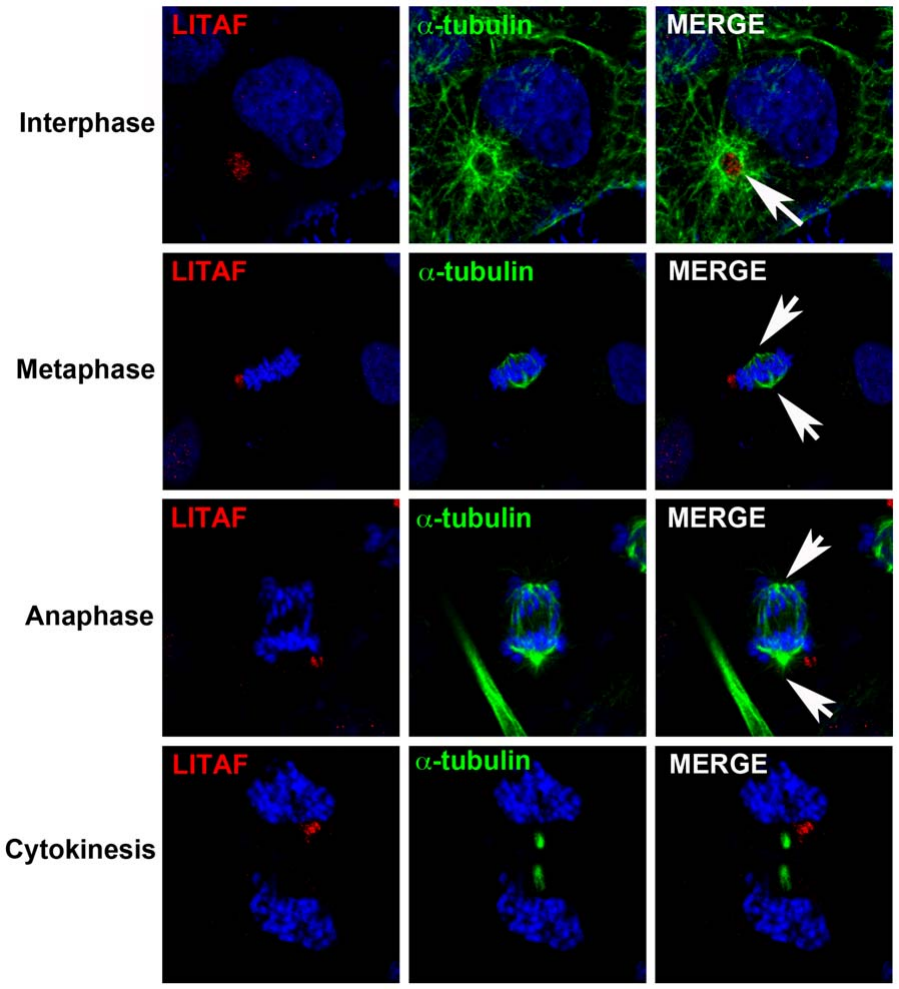
Endogenous LITAF undergoes asymmetric inheritance. In order to determine whether LITAF localized to centrosomes or aggresomes, BGMK cells were fixed and processed for indirect immunofluorescence. Cells were probed for endogenous LITAF (anti-LITAF antibodies; red), anti-α-tubulin (green), and ToPRO (Invitrogen; blue) were used to visualize the mitotic stage of each cell during interphase or mitosis. White arrows denote the sites of microtubule nucleation (MTOC).

Since the MTOC and aggresome are immediately adjacent to each other in the cell, it is difficult to discriminate whether endogenous LITAF is targeted to the MTOC or the aggresome. One method to discriminate between the MTOC and aggresome is to look at LITAF localization during mitosis. During mitosis, the centrosomes duplicate and move to the poles of the cell to segregate the chromosome pairs during metaphase. However, the aggresome does not divide and remains as a single structure in dividing cells that is inherited by one of the daughter cells [12]. In the daughter cell that does not inherit an aggresome, a new aggresome will eventually form as proteins destined for degradation begin to accumulate in the cell. Therefore, in order to clarify whether LITAF localized to aggresomes or centrosomes, anti-LITAF antibodies were used to visualize LITAF in BGMK cells. An α-tubulin antibody was used to visualize the cellular microtubule network during interphase and cell division. During interphase, LITAF was localized at the center of the α-tubulin network (Figure 2). However, during metaphase and anaphase of mitosis LITAF was localized away from the mitotic spindle (Figure 2). Finally, as the cells divide during cytokinesis, LITAF is inherited by only one daughter cell (Figure 2). These results suggest that LITAF is localized to aggresomes and not centrosomes. During cell division, two centrosomes exist, one at each pole of the cell. Therefore, if LITAF were associated with centrosomes, then one would expect LITAF staining at two separate locations. Aggresomes on the other hand are only inherited by a single daughter cell [12]. The asymmetrical inheritance of the aggresomes to only one cell generates different cell fates and is thought to be of evolutionary advantage [12]. One cell remains free of aggregated protein and any disease phenotype the aggregated proteins cause. The asymmetric inheritance of LITAF therefore suggests localization in aggresomes.

### The simple-like domain (SLD) is responsible for targeting LITAF to the aggresome

**Figure 3:**
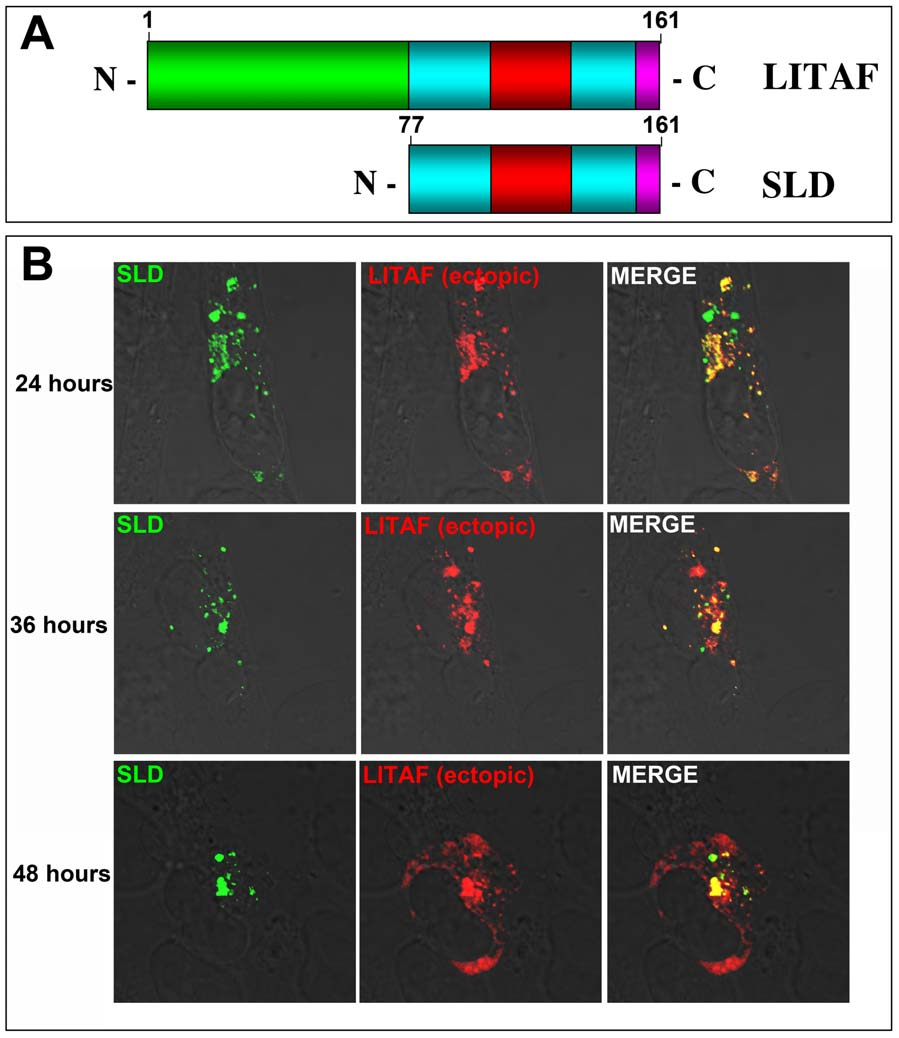
Over-expression of the SLD results in perinuclear localization. (A) Schematic of full-length LITAF and the SLD showing the proline rich N-terminus of LITAF (green) and the SLD is composed of a RING finger-like domain (cyan) which is interrupted with a hydrophobic domain (red). (B) BGMK cells were transiently co-transfected with FLAG-LITAF and myc-SLD. Twenty-four, 36, and 48 hours after transfection, cells were fixed and underwent indirect immunofluorescence. FLAG-LITAF was detected using an anti-FLAG antibody (red) and myc-SLD was detected using anti-myc antibodies (green). Yellow shows regions where the two proteins co-localize.

The SLD is a unique domain found in the C-terminus of LITAF. It contains a RING finger domain interrupted by a stretch of hydrophobic amino acids and the function of this domain remains elusive [1]. In order to determine whether the SLD alone was sufficient to target LITAF to aggresomes, we created two LITAF constructs. The first construct represents a full-length version of LITAF that was N-terminal FLAG-tagged. The Flag-tag allows us to detect the transfected LITAF without detecting endogenous LITAF. The second construct is a truncation of LITAF composed of only the C-terminus of LITAF, which we term the SLD (Figure 3A). The SLD construct was N-terminal myc-tagged. BGMK cells were transfected with either full-length LITAF or the SLD of LITAF. At 24 hours post-transfection, both LITAF and SLD showed a punctate staining in the cytoplasm of the cell with a high degree of co-localization (Figure 3B). We have previously shown that transfected LITAF localized to the late endosome/lysosome (Eaton et al., 2011). At 36 hours post-transfection, unlike full length LITAF, SLD staining appeared to redistribute within the cell, and became more concentrated a perinuclear location (Figure 3B). Some co-localization still existed between the SLD and LITAF, although it is less than that observed at 24 hours post-transfection. At 48 hours post-transfection, the SLD staining continued to concentrate in a perinuclear region (Figure 3B). Although patches of expression of SLD where still seen outside of the perinuclear region with regions that overlapped with full-length LITAF. It should be noted that most of the full-length LITAF staining did not overlap with the SLD.

**Figure 4:**
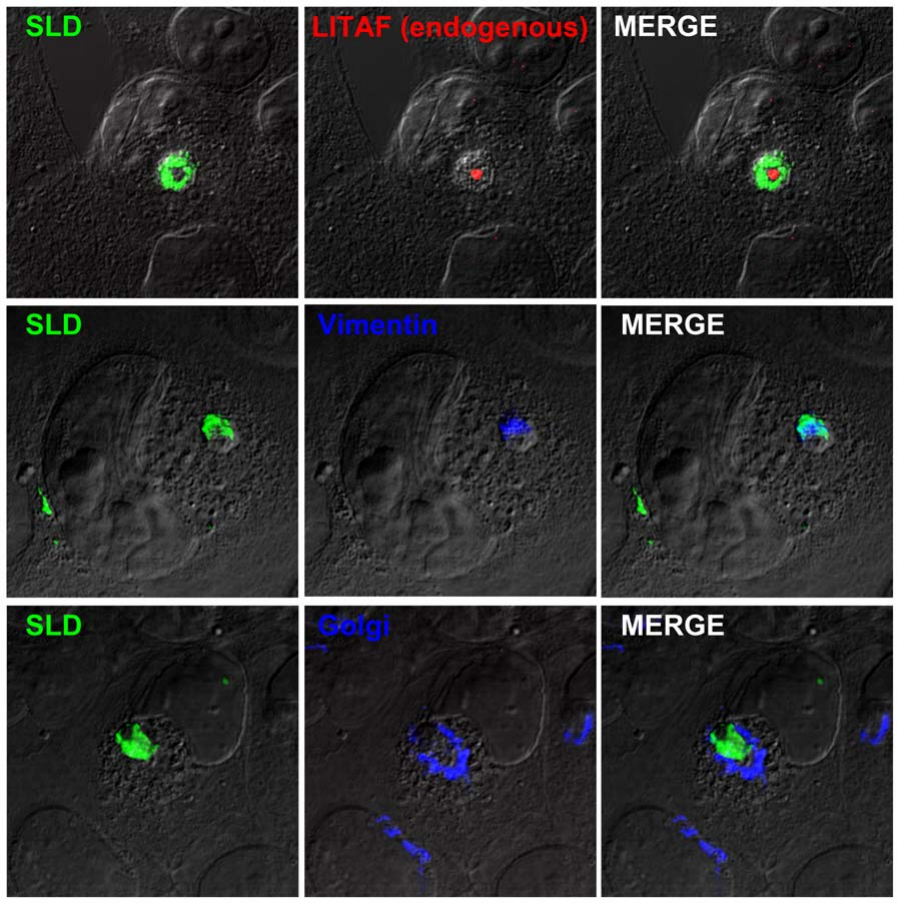
The SLD localizes to the aggresome. BGMK cells were transfected with myc-SLD. Forty-eight hours post-transfection, cells were fixed and immunofluorescence was completed with a variety of cellular markers in order to determine localization of the SLD (green). Endogenous LITAF was detected using an anti-LITAF antibody (red) and vimentin (anti-vimentin antibodies) and the Golgi apparatus (anti-Golgin 97) are visualized in blue. DIC was used to visualize the cell nuclei.

Since the SLD over time localizes to a small perinuclear region we decided to test whether the SLD was being targeted to the aggresome. By 36 hours, the SLD appears to become less associated with LITAF-positive compartment and more with another structure in the cell. In order to determine where within the cell the SLD was localizing to, the SLD construct was transfected into cells and a variety of cellular markers were examined. We found that the SLD staining coalesced around endogenous LITAF and vimentin staining (Figure 4), suggesting that the SLD is either forming around the aggresome or causing an expansion of the aggresome. We also demonstrated that the Golgi apparatus rearranged to form around SLD staining (Figure 4), which is consistent with other studies that found the Golgi apparatus surrounding aggresomes [9]. These results suggest that overtime the SLD construct changes localization within the cell and begins to converge in aggresomes, a site where endogenous LITAF is localized. In addition, the localization of either endogenous or ectopic LITAF, or the SLD was not affected by the presence of the proteasome inhibitor MG132 (data not shown).

## Discussion

The accumulation of protein aggregates is commonly linked with disease-associated mutant misfolded proteins including huntingtin [13], parkin [14] and peripheral myelin protein 22 (PMP22) [15]. Specifically, mutations or duplication of PMP22 increases its rate of misfolding and accumulation in aggresomes, resulting in Charcot-Marie Tooth (CMT) disease subtype 1A [15], [16], [17]. CMT is the most common group of inherited neuropathies that result in muscle weakness and progressive wasting [18]. There is extensive genetic heterogeneity in CMT with over 20 causative genes identified, including LITAF [19], [20], [21], [22], [23], [24], [25], [26], [27], [28]. Mutations in LITAF were recently identified as the molecular basis of CMT subtype 1C [29], [30]. However, at least one of the CMT mutations does not alter the ecotopic localization of LITAF [3] suggesting that the mutations may affect other aspects of LITAF function.

Mutations associated with CMT tend to cluster within the CXXC knuckles that form the consensus sequence of the SLD. This suggests that part of LITAF function resides in these regions. However, the mechanism involved in how LITAF causes CMT subtype 1C is unknown. It is currently unclear whether defects in LITAF function may result in an inappropriate accumulation of target protein, as is the case for PMP22. The fact that endogenous LITAF localizes to aggresomes suggests that wild-type LITAF may itself exhibit high levels of misfolding, possibly because of the highly hydrophobic nature of the SLD. In addition, the accumulation of misfolded proteins in the aggresome can lead to apoptosis [31]. Mutations throughout the SLD of LITAF may exaggerate this affect, completely overwhelming the degradation machinery of the cell resulting in the accumulation of aggregated LITAF protein. The possibility also exists that LITAF plays a specific function in the aggresome, although there is currently no evidence to support this.

Although LITAF localizes to aggresomes in BGMK cells, it is possible that this localization is cell-type specific. We were unable to observe endogenous LITAF in HEK-293, Hela, and primary neuronal cell lines. One explanation for our inability to observe endogenous LITAF in these cells is that LITAF maybe expressed at levels below our ability to detect LITAF by indirect immunofluorescence. Alternatively, LITAF appears to be upregulated in response to bacterial cell wall components [32], [33], [34]. So endogenous levels may remain undetectable until the cells are stimulated.

There appears to be a discrepancy between the localization of endogenous and recombinant LITAF within a cell. Recombinant LITAF exhibits localization with late endosomes/lysosomes [1], while we show for the first time here that endogenous LITAF accumulates in aggresomes and this accumulation may be mediated by the SLD in BGMK cells. One possibility for the discrepancy in cellular localization between endogenous and recombinant LITAF is over-expression of the recombinant construct. If endogenous LITAF accumulates in aggresomes then over-expression of LITAF may trigger a catabolic process called autophagy. Autophagy is mediated by an ubiquitin-like conjugative system and intracellular material is delivered to lysosomes for bulk degradation [35]. Autophagy is often used as a method by cells to dispose of aggresomes and avoid the unnecessary aggresome enlargement and saturation of degradation machinery [36], [37], [38]. Over-expression of LITAF would therefore result in its localization to lysosomes. It would be interesting to determine the rate of degradation of LITAF in cells (in the presence and absence of lysosome or proteasome inhibitors) to reveal whether this is the case.

It is also possible that ectopically expressed LITAF is also found in aggresomes. Ectopically expressed LITAF appears to be present in vesicles distributed throughout the cell that we have previously identified as the late endosome/lysosome [2], [3], [4]. In addition, ectopically expressed LITAF partially localizes with the SLD at 36 and 48 hours post transfection in all cells examined (Figure 3B). Since the SLD is localized with vimentin in the aggresome at 48 hours (Figure 4), this suggests that ectopic LITAF may also be localized, in part, to the aggresome. However, the bulk of the ectopic LITAF is localized to the late endosome/lysosome.

This paper describes for the first time a novel cellular localization for endogenous LITAF. We found using a variety of cellular markers that endogenous LITAF accumulates in aggresomes. This cellular localization of LITAF was also supported by the observation that LITAF undergoes asymmetric inheritance during mitosis. The process of asymmetrical inheritance confers an evolutionary advantage to one daughter cell. Furthermore, we found that the C-terminus of LITAF may mediate this localization. The role of LITAF in CMT further stresses the importance of understanding how LITAF functions and what role the aggresome plays in the development of this disease.

## Materials and Methods

### Reagents, cell lines, and antibodies

Baby green monkey kidney (BGMK) cells were obtained from the American Type Culture Collection (ATCC; Manassas, VA) and were maintained at 37°C with 5% CO_2_ in Dulbecco's modified Eagle's medium (DMEM; HyClone, Ottawa, ON) supplemented with 7% FBS, 2 mM L-glutamine, penicillin (100 U/mL), and streptomycin (100 µg/mL). Cells used for immunofluorescence were transfected using a polyethylenimine (PEI) reagent using 5 µg plasmid/10 cm^2^ plate and a PEI:DNA ratio of 4∶1. Antibodies used during immunofluorescence include: mouse polyclonal anti-LITAF antibody (dilution 1/100, BD Biosciences, San Jose, CA); anti-Nedd4 antibodies (dilution 1/100, BD Biosciences, Mississauga, ON); α-tubulin antibody (dilution 1/100, Santa Cruz Biotechnology, Santa Cruz, CA); anti-myc monoclonal antibody 9E10 myc monoclonal antibody (dilution - 1/100, Roche, Indianapolis, IN); anti-FLAG (M2) monoclonal antibody (dilution 1/500, Sigma, Oakville, ON); anti-Golgin 97 (dilution 1/100, Invitrogen, Burlington, ON); and FITC/Cy3/Cy5-conjugated goat anti-mouse or anti-rabbit immunoglobulin G (IgG) from Jackson ImmunoResearch Inc. (dilutions – 1/100, 1/200, 1/100 respectively; West Grove, PA).

### Expression plasmids

Full length mouse LITAF and the SLD of LITAF were amplified using a PCR mixture containing 1X PCR buffer (Invitrogen, Burlington, ON), 3.0 mM MgCl_2_, 2.5 U Taq DNA polymerase (5 U/µL; Invitrogen), 0.2 mM forward primer, 0.2 mM reverse primer, and mouse LITAF cDNA (ATCC) as template DNA. LITAF was amplified and a FLAG tag was added to the N-terminus using the following forward and reverse primers: (LITAF-forward) 5′ – AAGCTTATGGATTACAAGGATGACGACGATAAGTCGGTTCCAGGACCTTACC - 3′, and (LITAF-reverse) 5′ – CTCGAGCTAAAAGCGTTGTAGGTG - 3′. The SLD was amplified and a myc tag was included at the N-terminus using the following forward and reverse primers: (SLD-forward) 5′ -ATGGAACAAAAACTTATTTCTGAAGAAGATCTGGTGCAGACGGTCTACGTGCAG - 3′ and LITAF-reverse (above). The following cycling conditions were used: 94°C for 30 seconds, 52°C for 30 seconds, 72°C for 90 seconds for 30 cycles. The resulting PCR constructs were initially cloned into the vector pGEM-T easy (Promega, Madison, WI), followed by cloning into the *XhoI* and *HindIII* sites of pcDNA3.1 (Invitrogen).

### Immunofluorescence analysis

Cells were fixed for ten minutes in 3.7% paraformaldehyde in PBS. Cells were washed several times in PBS and were permeabilized in a 0.1% Triton X-100 in PBS solution for four minutes. Following several washes in PBS, cells were blocked for two hours at room temperature in block buffer (5% BSA (w/v), 50 mM Tris HCl (pH 7.4), 150 mM NaCl, 0.5% NP-40 (v/v). Cells were then washed several times with wash buffer (1% BSA (w/v), 50 mM Tris HCl (pH 7.4), 150 mM NaCl, 0.5% NP-40 (v/v)) and were incubated for one hour at room temperature with primary antibody diluted in wash buffer. Cells were washed several times in wash buffer and incubated for one hour at room temperature in darkness with secondary antibody diluted in wash buffer. Following several more washes in wash buffer, fluorescence was detected using a Leica DM SP2 confocal microscope (Leica, Wetzlar, Germany) and images were assembled using Adobe Photoshop CS4 (Adobe, San Jose, CA).
